# Genetic polymorphisms in the vitamin D pathway in relation to lung cancer risk and survival

**DOI:** 10.18632/oncotarget.2951

**Published:** 2014-12-10

**Authors:** Jinyu Kong, Fangxiu Xu, Jinli Qu, Yu Wang, Ming Gao, Herbert Yu, Biyun Qian

**Affiliations:** ^1^ Tianjin Medical University Cancer Institute and Hospital, National Clinical Research Center of Cancer, Tianjin, China; ^2^ Department of Cancer Epigenetics Laboratory, First Affiliated Hospital of Henan University of Science and Technology, Luoyang, China; ^3^ Hongqiao International Institute of Medicine, Shanghai Tongren Hospital/Faculty of Public Health, Shanghai Jiao Tong University School of Medicine, Shanghai, China; ^4^ Epidemiology Program, University of Hawaii Cancer Center, Honolulu, HI, USA

**Keywords:** non-small cell lung cancer, vitamin D pathway, single nucleotide polymorphism, genetic susceptibility, prognosis

## Abstract

Studies have suggested that vitamin D may have protective effects against cancer development or tumor progression. To search for additional evidence, we investigated the role of genetic polymorphisms involved in the vitamin D pathway in non-small cell lung cancer (NSCLC). We evaluated common genetic polymorphisms associated with the vitamin D pathway in relation to NSCLC in a case-control study of 603 newly diagnosed NSCLC patients and 661 matched healthy controls. Seven single nucleotide polymorphisms (SNPs) were genotyped, the expression of *CYP27B1* and *CYP24A1* were measured in 153 tumor samples and their associations with genotypes and patient survival were also analyzed. In the case-control comparison, we found SNP rs3782130 (*CYP27B1*), rs7041 (*GC*), rs6068816 and rs4809957 (*CYP24A1*) associated with NSCLC risk. The risk of NSCLC was increased with the number of risk alleles. *CYP27B1* and *CYP24A1* expression were significantly different between tumor and normal tissues in NSCLC. High *CYP27B1* expression was associated with better overall survival, and the expression was different by the rs3782130 genotype. The study suggests that some genetic polymorphisms involved in the vitamin D pathway may associate with NSCLC risk, and one of the polymorphisms (rs3782130) may affect gene expression and patient survival.

## INTRODUCTION

Non-small cell lung cancer (NSCLC) is one of the most frequently diagnosed malignancies as well as the leading cause of cancer death worldwide [1, 2]. Research has shown that environment, lifestyle and genetic factors may act jointly in the development of NSCLC. Risk factors associated with environment and lifestyle include cigarette smoking, air pollution, history of lung diseases, nutrition, and exposure to radon, asbestos and radiation [3, 4]. Studies also suggest that genetic variations and alterations may play a role in lung cancer in addition to environmental factors and lifestyles [5]. Numerous oncogenes, tumor suppressor genes and DNA repair genes have been linked to the pathogenesis of NSCLC [6].

Vitamin D is known to have functions extended beyond bone formation that include enhancing immune function and suppressing cell proliferation [7]. Furthermore, vitamin D may inhibit tumor progression and reduce the mortalities of several malignancies, including lung cancer [8, 9]. Vitamin D has also been reported to have anti-proliferative, anti-angiogenic, anti-metastatic, and pro-apoptotic effects [10-12]. These characteristics indicate that vitamin D may be used as a potential chemopreventive agent to treat hyper-proliferative disorders and prevent the development of cancer.

Bioactive vitamin D is synthesized through a series of reactions catalyzed by a number of enzymes. *CYP2R1* and *CYP27A1*, which are 25-hydroxylases, first convert pro-vitamin D absorbed from diet or produced in the skin after sun exposure to a major circulating form of vitamin D, 25(OH)D. Next, *CYP27B1*, 1a-hydroxylase converts 25(OH)D to 1,25-dihydroxyvitamin D [1,25(OH)_2_D_3_] either in the kidney (where it is released into the circulation) or specific target organs. Circulating 1,25(OH)_2_D_3_ is degraded by CYP24A1. Both vitamin D metabolites bind to the vitamin D-binding protein, also known as group-specific component (*GC*), which facilitates vitamin D transportation. In target tissues, 1,25(OH)_2_D_3_ binds to the vitamin D nuclear receptor (*VDR*). The complex further forms a heterodimer with the retinoid X receptor (*RXR*), and the heterodimer binds to vitamin D response elements on multiple genomic loci, some of which are known to possess anticancer properties [13]. Genetic polymorphisms involved in the vitamin D pathway may affect its activities, and therefore may be related to lung cancer if vitamin D indeed plays a role in the disease.

To investigate vitamin D-related genetic polymorphisms in association with NSCLC, we conducted a case-control study. In the study, we selected single nucleotide polymorphisms (SNPs) located in the protein coding or promoter regions from tagging SNPs, including rs3782130 and rs10877012 in *CYP27B1*, rs6068816 and rs4809957 in *CYP24A1*, rs11574129 in *VDR*, rs7041 in *GC*, and rs10741657 in *CYP2R1*. In addition to the analysis of risk associations with individual SNPs, we also evaluated gene-gene and gene-environment interactions, and examined the effects of SNPs on mRNA expression and their associations with NSCLC survival.

## RESULTS

### Subject characteristics

The study included 603 NSCLC cases and 661 frequency-matched healthy controls. The average age of study participants was 60 years, ranging from 23 to 83 years. There were no statistically significant differences in the distributions of sex, gender and history of pulmonary diseases between patients and controls. However, there were significant differences between cases and controls in family history of cancer, smoking status and BMI. As expected, more lung cancer patients were smokers compared to the controls (66.5% vs. 42.2%, p < 0.001). Lung cancer patients were also more likely to have a family history of cancer than the controls (17.1% vs. 9.2%, p<0.001). Detailed characteristics of the study population are shown in Table 1.

**Table 1:** Characteristics of NSCLC patients and healthy controls

Variable	Number of subject(%)		P Value
	Case (n=603)	Control (n=661)	
Age (n=1264)			0.433
<60	297(49.3)	311(47.0)	
≥60	306(50.7)	350(53.0)	
Gender (n=1264)			0.439
Male	387(64.2)	410(62.0)	
Female	216(35.8)	251(38.0)	
History of pulmonary disease (n=1214)			0.789
No	540(89.6)	550(90.0)	
Yes	63(10.4)	61(10.0)	
Family history of cancer (n=1255)			**<0.001**
No	498(82.9)	594(90.8)	
Yes	103(17.1)	60(9.2)	
Smoking status (n=1264)			**<0.001**
No	202(33.5)	382(57.8)	
Yes	401(66.5)	279(42.2)	
BMI (n=1248)			**<0.001**
<24	279(46.7)	227(34.9）	
≥24	318(53.3)	424(65.1)	

### Associations of SNPs and NSCLC

Seven SNPs in five genes implicated in the vitamin D pathway were examined in the study. Table 2 shows the genotype frequency of each polymorphism in the cases and controls and their corresponding odds ratios (OR) after adjustment for age, gender, cigarettes smoking, family history of cancer and BMI. Three genetic models, including co-dominant, dominant, and recessive, were utilized to calculate the OR. Comparing the genotypes between cases and controls, we found SNPs rs3782130 (*CYP27B1*), rs6068816 (*CYP24A1*), rs4809957 (*CYP24A1*) and rs7041 (*GC*) associated with lung cancer risk, and the associations included the G/G genotype of rs3782130 (adjusted OR=1.60; 95% CI: 1.09-2.34), T/T genotype of rs6068816 (adjusted OR=0.40; 95% CI: 0.26-0.60), A/A genotype of rs4809957 (adjusted OR=2.32; 95% CI: 1.40-3.87), and G/G genotype of rs7041 (adjusted OR=0.57, 95% CI: 0.35-0.93).

**Table 2:** Associations between NSCLC risk and individual SNPs involved in the vitamin D pathway

Genotype	Number of subject (%)		P	OR^a^(95%CI^b^)	OR^a^(95%CI^b^) ②+③ vs. ①	OR^a^(95%CI^b^) ③ vs. ①+②
	Case (n=603)	Control (n=661)				
CYP27B1 (rs3782130)						
CC	229(38.0)	230(34.8)	**0.022**	1.00	1.00	1.00
CG	297(49.3)	371(56.1)		0.82(0.63-1.06)	0.90(0.70-1.15)	**1.60(1.09-2.34)**
GG	77(12.8)	60(9.1)		1.42(0.94-2.14)		
CYP27B1 (rs10877012)						
TT	235(39.0)	243(36.8)	0.331	1.00	1.00	1.00
TG	273(45.3)	326(49.4)		0.87(0.67-1.13)	0.92(0.72-1.17)	1.19(0.85-1.66)
GG	94(15.6)	91(13.8)		1.10(0.76-1.58)		
CYP24A1 (rs6068816)						
CC	217(36.0)	110(16.6)	**<0.001**	1.00	1.00	1.00
CT	314(52.1)	465(70.3)		**0.33(0.25-0.44)**	**0.34(0.26-0.45)**	0.87(0.61-1.24)
TT	72(11.9)	86(13.0)		**0.40(0.26-0.60)**		
CYP24A1 (rs4809957)						
GG	234(38.8)	228(34.5)	**<0.001**	1.00	1.00	1.00
GA	309(51.2)	406(61.4)		**0.77(0.60-0.99)**	0.88(0.69-1.12)	**2.71(1.66-4.41)**
AA	60(10.0)	27(4.1)		**2.32(1.40-3.87)**		
VDR (rs11574129)						
TT	339(56.2)	378(57.2)	0.873	1.00	1.00	1.00
TC	254(42.1)	274(41.5)		1.06(0.84-1.35)	1.07(0.84-1.36)	1.36(0.50-3.70)
CC	10(1.7)	9(1.4)		1.39(0.51-3.81)		
GC (rs7041)					
TT	329(54.6)	272(41.1)	**<0.001**	1.00	1.00	1.00
TG	240(39.8)	339(51.3)		**0.59(0.46-0.76)**	**0.59(0.46-0.75)**	0.73(0.46-1.18)
GG	34(5.6)	50(7.6)		**0.57(0.35-0.93)**		
CYP2R1 (rs10741657)						
GG	252(41.9)	246(37.2)	0.216	1.00	1.00	1.00
GA	270(44.9)	326(49.3)		0.82(0.64-1.06)	0.83(0.65-1.06)	0.96(0.68-1.36)
AA	80(13.3)	89(13.5)		0.86(0.59-1.25)		
Number of risk-allele						
0	138(22.9)	299(45.2)		1.00		
1	282(46.8)	267(40.4)		**2.46(1.86-3.25)**		
2	147(24.4)	83(12.6)		**4.25(2.96-6.12)**		
3	35(5.8)	12(1.8)		**5.80(2.84-11.84)**		

a OR: Odds Ratio adjusted by age, sex, family history of cancer and BMI.

b CI: Confidence Interval.

① Homozygous wild genotype, ② heterozygous genotype, ③ homozygous variant genotype

### Effect of multiple polymorphisms

To test if multiple SNPs in the vitamin D pathway have a combined effect on lung cancer risk, we analyzed the four significant SNPs together. Based on the results above, we assigned numbers of risk alleles in rs3782130, rs6068816, rs4809957 and rs7041 for each individual, and analyzed the association of risk allele numbers with NSCLC risk. A significant trend was observed between numbers of risk alleles and lung cancer risk. With increasing numbers of risk alleles, the risk of NSCLC was significantly elevated (Table 2). Compared to those without any risk-allele, subjects with three risk alleles had more than 5-fold increases in risk of NSCLC (OR=5.80, 95%CI: 2.84-11.84).

### Smoking-gene interaction

When the data were analyzed in subgroups of subjects stratified by smoking status, we found that smokers with variant homozygous rs3782130 in *CYP27B1* had significantly increased risk of lung cancer (OR=1.91; 95% CI: 1.04-3.53). Nonsmokers and smokers with variant homozygous rs4809957 in *CYP24A1* had higher risk than those with other genotypes (OR=2.33; 95% CI: 1.20-4.53 and OR=3.49; 95% CI: 1.63-7.47) (Table 3). Logistic regression analyses showed significant joint effects between smoking and *CYP27B1* (rs3782130) or *CYP24A1* (rs4809957) (p<0.001).

**Table 3:** Associations between NSCLC risk and SNPs by smoking status

Genotype	Non-smoker			Smoker		
	Cases (%)	Controls (%)	OR^a^(95%CI^b^)	Cases (%)	Controls (%)	OR^a^(95%CI^b^)
CYP27B1 (rs3782130)						
CC+CG	170(84.2)	338(88.5)	1.00	356(88.8)	263(94.3)	1.00
GG	32(15.8)	44(11.5)	1.38(0.83-2.30)	45(11.2)	16(5.7)	**1.91(1.04-3.53)**
P Value	0.140			**0.014**		
CYP24A1 (rs4809957)						
GG+GA	181(89.6)	364(95.3)	1.00	362(90.3)	270(96.8)	1.00
AA	21(10.4)	18(4.7)	**2.33(1.20-4.53)**	39(9.7)	9(3.2)	**3.49(1.63-7.47)**
P Value	**0.009**			**0.001**		

a OR: Odds Ratio adjusted by age, sex, family history of cancer and BMI.

b CI: Confidence Interval.

### Expression of CYP27B1 and CYP24A1

**Figure 1:**
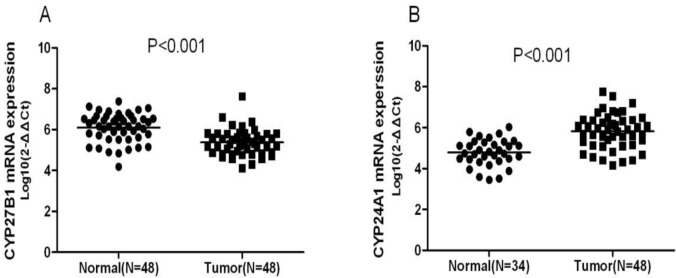
RT-qPCR results on *CYP27B1* and CYP24A1 expression in lung tumor (n =48) and non-tumor tissues (n =48) Compared to non-tumor tissues, *CYP27B1* mRNA expression was significantly lower in tumor tissues (p<0.001) (Figure 1A), and the expression of *CYP24A1* was significantly higher in tumor tissues (p<0.001) (Figure 1B).

In this study, we found possible associations between NSCLC and SNPs rs3782130 (*CYP27B1*), rs6068816 (*CYP24A1*), rs4809957 (*CYP24A1*) or rs7041 (*GC)*. SNPs rs3782130 and rs4809957 are located in the 5′ end of the *CYP27B1* gene and the 3′ UTR region of the *CYP24A1* gene, respectively. These locations may affect the transcription of a target gene and the stability of its mRNA. Based on the understandings, we performed RT-qPCR to assess the expression of *CYP27B1* and *CYP24A1* in lung tumor and non-tumor tissues. Compared to non-tumor tissues, *CYP27B1* mRNA expression was significantly lower in tumor tissues (p<0.001) (Figure 1A), and the expression of *CYP24A1* was significantly higher in tumor than in non-tumor tissues (p<0.001) (Figure 1B).

### CYP27B1 and CYP24A1 expression and NSCLC survival

**Figure 2:**
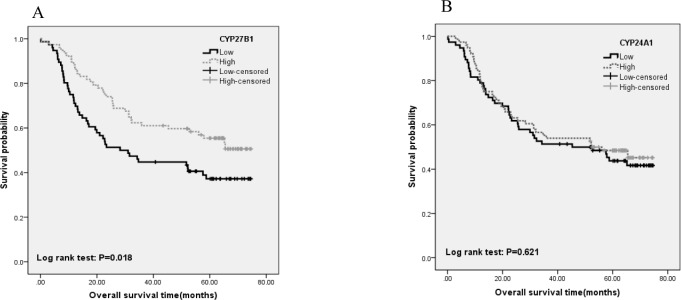
Associations of *CYP27B1* and *CYP24A1*expression with overall survival of 153 NSCLC patients Kaplan-Meier survival analysis showed that patients with high *CYP27B1* expression had better overall survival than those with low *CYP27B1* (p=0.018, Figure 2A). No significant association was found between *CYP24A1* expression and NSCLC survival (p=0.621, Figure 2B).

Levels of *CYP27B1* and *CYP24A1* expression in tumor tissues were analyzed in 153 NSCLC patients. Based on the median expression, patients were categorized into high and low expression groups. Kaplan-Meier survival analysis showed that patients with high *CYP27B1* expression had better overall survival than those with low *CYP27B1* (p=0.018, Figure 2A). No significant association was found between *CYP24A1* expression and NSCLC survival (p=0.621, Figure 2B).

Univariate and multivariate Cox regression analyses were also carried out to evaluate the associations of gene expression and survival outcome. The results of univariate analysis showed that high *CYP27B1* expression in tumors was significantly associated with better overall survival (p=0.020). However, this association was attenuated after covariates and confounding variables were adjusted in the regression model (HR=0.79; 95%CI: 0.50-1.26). For older patients (age>60 years), high *CYP27B1* expression seemed to remain the significant association with better NSCLC survival (HR=0.51, 95%CI: 0.26-0.98). Similar to Kaplan-Meier survival analysis, no significant association was found between *CYP24A1* expression and NSCLC survival (Supplement table 1).

### SNP associations with expression in CYP27B1 and CYP24A1

To examine if the SNPs in *CYP27B1* and *CYP24A1* affect their mRNA expression, we compared levels of mRNA expression by the genotypes of *CYP27B1* and *CYP24A1* in 153 tumor samples. The results showed that *CYP27B1* expression was significantly different by the genotype of rs3782130 (p=0.028). Expression in the G/G genotype of rs3782130 was lower than that of the C/C genotype. No difference in expression, however, was found for the genotype of *CYP24A1* (Table 4).

**Table 4:** Expression of CYP24A1 and CYP27B1 by their SNP genotypes

Gene	Genotype	Case (n=153)	Mean ± SD	P value
CYP27B1(rs3782130)				**0.028**
	CC	53	5.60±0.66	
	CG	81	5.41±0.66	
	GG	19	5.14±0.58	
CYP24A1(rs4809957)				0.316
	GG	56	5.64±0.76	
	GA	88	5.69±0.96	
	AA	8	6.15±0.79	

## DISCUSSION

Observational studies have shown that lung cancer incidences in certain populations are correlated with serum levels of vitamin D. A Finnish study found an inverse association between serum 1,25(OH)_2_D_3_ and lung cancer risk in women or those under the age of 50 years; no association was seen in men or older people[14]. Furthermore, in a US study of 456 patients with early-stage NSCLC, Zhou et al. demonstrated that improved survival in patients with early stage lung cancer was associated with higher circulating levels of 1,25(OH)_2_D_3_[15, 16]. These observations suggest that vitamin D may play an important role in the development and progression of lung cancer.

*CYP27B1* catalyzes the second 1a-hydroxylation of 25(OH)D in the kidney or some extra-renal tissues to produce the active form of vitamin D, 1,25-dihydroxyvitamin D_3_. Compared to epithelial cells in the normal airway, some small cell and non-small cell cancer cell lines express very low or no *CYP27B1*[17, 18]. Increased expression of *CYP27B1* has been reported in alveolar macrophages of lung cancer patients, with the highest expression being found in more advanced disease stages[19]. In this study, we found *CYP27B1* expression was lower in lung cancer than in normal tissues, and the patients with high *CYP27B1* expression had better survival outcome, especially in old patients. SNP rs3782130 is located in the *CYP27B1* promoter. Promoters are important cis-acting elements that regulate gene expression. SNPs in the region may influence transcription and have impacts on gene function [20]. Holt et al found a significant association between rs3782130 and prostate cancer relapse and death[21]. Our study showed that *CYP27B1* expression in lung cancer was different by the rs3782130 genotype. The genotype was related to the risk of lung cancer, and the expression was associated with survival. Furthermore, we analyzed several public databases, including SNPinfo, TFsearch and Transfac, and found that this SNP site may be a binding site for a transcription factor, nuclear factor 1 (*NF-1*) which is a site-specific DNA-binding protein and plays an important role in regulating the transcription of many genes[22, 23]. *NF-1* may activate or repress gene expression by binding to the promoter of a target gene. Moreover, *NF-1* can interact with various co-activators to regulate gene transcription. For example, binding of *NF-1/YY1* to the p53 promoter activates *p53* gene expression [24, 25], while loss of transcription repressor complex *NF-1/Rb/HDAC-1* was observed in human metastatic breast cancer cells[26]. We speculate that different genotypes in rs3782130 may interact with *NF-1* differentially, which leads to distinct regulatory effects on *CYP27B1* transcription.

*CYP24A1* is a member of the cytochrome P450 superfamily and an enzyme degrading 1,25(OH)_2_D_3_. Several studies have demonstrated that *CYP24A1* is overexpressed in lung tumors compared with normal lung tissues [27-29]. Chen et al. found that *CYP24A1* was highly expressed in poorly differentiated cancer and high expression was associated with poor patient survival, suggesting that *CYP24A1* is an independent prognostic marker for lung cancer [30]. These research findings indicate that *CYP24A1* may have tumor promoting effects. In our study, we also found that *CYP24A1* expression was significantly higher in lung tumors than in normal tissues. This observation is in agreement with the findings of previous studies, despite no significant association between *CYP24A1* expression and lung cancer survival in our study. SNP rs6068816 is a synonymous polymorphism which does not alter the amino acid sequence of *CYP24A1*, but may influence intron splicing. SNPs located in the enhancers or silencers of splicing regions may affect the patterns or efficiency of mRNA splicing, which in turn have impacts on phenotype or biologic activities [31]. Regarding another *CYP24A1* SNP, rs4809957, our finding suggests the variant genotype associated with increased risk of lung cancer. This observation is consistent with a previous GWAS report [32]. We did not find any differences in mRNA expression by the rs4809957 genotype. SNP rs4809957 locates in the 3′ untranslated region of *CYP24A1*, and has no known functional relevance or structural alteration in the *CYP24A1* protein. However, the SNP region is linked to a poly (A) microsatellite repeat that may be associated with the stability of *CYP24A1* mRNA.

As a key protein of vitamin D pathway, group-specific component (*GC*, also known as vitamin D-binding protein) can bind to vitamin D and its plasma metabolites, and transport them to target tissues. In addition, *GC* can be converted into a macrophage activating factor (*GcMAF*) through the stepwise reactions of β-galactosidase of B cells and sialidase of T cells. GcMAF has shown anti-tumor activities in mice [33] and is considered a potential immunotherapeutic agent for metastatic breast cancer [34]. SNP rs7041 was a common nonsynonymous SNP in the *GC* gene. Previous studies have investigated SNP rs7041 in relation to breast cancer [35-37], prostate cancer [38], gastrointestinal [39] and basal cell carcinomas [40]. However, to date, no systematic evaluation has been done on rs7041 with regard to its role in the development of lung cancer among Chinese. Our study suggests that the minor allele of rs7041 may be associated with reduced risk of NSCLC. Other studies have shown that the rs7041 genotype is associated with circulating 25(OH)D or 1,25(OH)_2_D_3_. Plasma levels of 25(OH)D was higher in people carrying the G/G genotype of rs7041 than those of the T/T carriers [41-43]. Taken together, these findings underscore the possibility that the decreased risk of NSCLC in the G/G carriers of SNP rs7041 may be due to its influence on vitamin D.

Although our study showed a number of interesting findings which seem to be consistent with existing knowledge or previous reports, we must be cautious to interpret our results because of several inherited limitations in our study. First, as a hospital-based case-control study, we cannot rule out the possibility of selection bias for our study subjects. Second, our study findings on genetic polymorphisms may apply only to the ethnicity involved in this study. Third, we did not measure serum or plasma levels of 25(OH)D and other known 25(OH)D which are the most relevant functional molecules. Fourth, we did not take into account of dietary supplement of vitamin D which can be an important confounding factor. Fifth, concerning the associations of genetic polymorphisms, the study is still lack of power. More studies with larger sample size are needed to validate these results.

In summary, we observed statistically significant associations between NSCLC and SNPs in *CYP27B1* (rs3782130), *CYP24A1* (rs6068816, rs4809957) and *GC* (rs7041). Furthermore, we found that SNP rs3782130 had a possible influence on *CYP27B1* expression and the expression was associated with the prognosis of NSCLC. These findings, though require further confirmation from large independent studies, seem to support the notion that vitamin D has a beneficial effect on lung cancer.

## MATERIALS AND METHODS

### Study subjects

Patients diagnosed with histologically confirmed primary NSCLC were recruited for the study at the Tianjin Medical University Cancer Hospital (TMUCH) during January 2006 through May 2011. Clinical information collected for the study includes histological type, tumor size, lymph node metastasis, and disease stage. These patients were also followed from surgery to August 20, 2013 though clinical visits and regular telephone contacts. Survival time was calculated from the date of diagnosis to the date of death or last follow-up (August 20, 2013). The control subjects were recruited from those who underwent regular health check-up during the same time as the cases being recruited. The controls lived in the same neighborhoods as the cases or nearby communities, and were frequency-matched to the cases on gender and age. All study subjects were genetically unrelated Han ethnic Chinese.

The study was approved by the ethical review committee at TMUCH. Each study participant signed an informed consent, and completed an in-person interview conducted by trained research staff of the study using a structured questionnaire. The questionnaire elicits information on demographic features, family history of cancer, history of pulmonary disease and tobacco use. Smoking status, age at first use, years of smoking, number of cigarettes smoked per day, and the status and age of quitting smoking were asked during the interview. Individuals who smoked one cigarette or more a day for at least half a year were considered smokers. A blood sample (10 ml) was collected from each study subject using an ethylene diamine tetraacetic acid (EDTA) vacutainertube. Plasma was separated after centrifugation at 2,000 rpm for 20 min and then put into a liquid nitrogen tank for long-term storage DNA extraction and genotyping. All matched tissue samples were histologically confirmed. These tissue samples were stored at −80°C until analysis.

### SNP selection and genotyping

Using the Tagger algorithm in the HaploView program and dense genotyping data from the International HapMap Phase III CHB samples, we identified tagging SNPs with linkage disequilibrium (LD) greater than 0.8 in the five selected genes (*CYP27B1, CYP24A1, VDR, GC,* and *CYP2R1*) plus the regions 100 kb up- and downstream of each gene. In addition, we selected SNPs located in the protein coding or promoter regions from tagging SNPs. When multiple SNPs are present in each genomic region, selection priority was given to those with known function or being previously investigated by other studies. Selection was also restricted to those with minor allele frequency >5% in the reference.

The selected genetic polymorphisms were genotyped with the polymerase chain reaction (PCR)-based fluorescence 5′ nuclease assay (TaqMan). In the PCR reaction, 30ng of genomic DNA were mixed with a PCR cocktail that contained two fluorescence-labeled allele specific probes (200nM each), forward and reverse primers (1μM each), and 2.5μL of 2×TaqMan Universal PCR Master Mix containing ROX passive dye as internal control, dNTPs, PCR buffer, and Taq polymerase (Applied Biosystems, Foster City, CA) in a final volume of 5μL. PCR amplification was completed under the conditions of 1 cycle of denaturing at 95°C for 10 min and 45 cycles of denaturing at 92°C for 15 sec and annealing at 60°C for 1 min, followed by storing at 4°C. The fluorescence of PCR products was plotted, and the genotypes were determined using the allelic discrimination software. Samples without DNA template and with known genotypes were included in each plate to monitor genotyping quality. Five percent of the samples selected randomly were retested, and the results were in complete concordance. All TaqMan assays were run in the ABI 7500 thermal cycle (Applied Biosystems, Foster City, CA).

### RNA extraction and RT-qPCR

Total RNA was extracted from flash frozen tumor samples using the TRizol reagents (Applied Biosystems, Foster City, CA) following the manufacturer's instructions. The quality and quantity of total RNA were determined with the Nanodrop N-1000 (Agilent Biosystems TM, Santa Clara, CA). All RNA samples were standardized to 50ng/μL before reverse transcription. The reverse-transcriptase (RT) reaction was carried out using the TaqMan mRNA Reverse Transcription kit (Applied Biosystems, Foster City, CA) based on the manufacturer's instructions. RT reaction was processed at 25°C for 10 min, 37°C for 50 min, and 70°C for 15 min. Following the RT, quantitative PCR (qPCR) was performed in the ABI 7900 Real-Time PCR system (Applied Biosystems, Foster City, CA). The PCR reaction began with incubation at 95°C for 10 min, and was followed by 40 cycles of 95°C for 15 sec and 60°C for 1 min. The PCR cycle threshold (Ct) was determined by the SDS 2.4 software (Applied Biosystems, Foster City, CA). Each sample was analyzed in a final volume of 20μL containing approximately 100ng of cDNA. Glyceraldehyde 3-phosphate dehydrogenase (*GAPDH*) expression was used as reference to standardize the *CYP27B1* and *CYP24A1* results. Relative levels of gene expression were calculated using the formula, 2^−ΔΔCt^.

### Statistical analysis

Chi-square test was used to compare the frequency distributions between cases and controls of demographic variables, environmental factors and gene polymorphisms. For continuous variables, differences were compared by analysis of variance (ANOVA) or Student t-tests. Hardy-Weinberg equilibrium was calculated for each SNP in the control subjects. Unconditional logistic regression model was used to examine the association between SNPs and lung cancer risk. In the logistic analysis, odds ratios (ORs) and their 95% confidence intervals (95% CI) were calculated with adjustment for confounding factors. Subgroup analyses were also performed for each polymorphism by smoking status. RNA expression was analyzed both as continuous and categorical variables. In categorical analysis, RNA expression was grouped into low and high categories using the median as cutoff. Survival curves were generated using the Kaplan-Meier method. Log-rank test and Cox proportional hazards regression model were used to compare differences in overall survival. All reported P values are two-sided. Results were considered statistically significant when p value was less than 0.05. All statistical analyses were accomplished using the SPSS version 17.0 (SPSS Inc., Chicago, IL) and Graphpad Prism 5.0 (Graphpad Software Inc., La Jolla, CA).

## SUPPLEMENTARY TABLE


