# Supplementary material for: Genetic polymorphisms in the vitamin D pathway in relation to lung cancer risk and survival
Source: Oncotarget. 2014 Dec 10;6(4):2573–82. doi: 10.18632/oncotarget.2951 (PMC4385872; doi:10.18632/oncotarget.2951)
Supplement: Supplementary file 1 [file oncotarget-06-2573-s001.pdf]

Genetic polymorphisms in the vitamin D pathway in relation to lung cancer risk and survival

Supplementary Material

Supplement table 1: **Associations between NSCLC survival and expression of CYP27B1 and CYP24A1**

	Overall Survival		HR[a] (95%CI[b])	
Factor	HR[a] (95%CI[b])	P value	Age≥60	Age<60
<u>Univariate analysis</u>	n=153			
CYP27B1 mRNA (High vs. Low）	**0.60(0.39-0.92)**	**0.020**	**0.48(0.26-0.88)**	0.74(0.40-1.36)
CYP24A1 mRNA (High vs. Low）	0.90(0.58-1.38)	0.621	0.92(0.50-1.70)	0.87(0.47-1.62)
<u>Multivariate analysis</u>	n=153			
CYP27B1 mRNA (High vs. Low）	0.79(0.50-1.26)	0.327	**0.51(0.26-0.98)**	1.27(0.61-2.67)
Age (≥60 vs.<60）	1.26(0.80-1.98)	0.314	-	-
Gender（Female vs. Male)	0.88(0.55-1.41)	0.603	1.45(0.66-3.16)	0.92(0.441.91)
Disease stage (Ⅲ-Ⅳ vs. Ⅰ-Ⅱ)	**5.59(3.44-9.08)**	**<0.001**	**7.89(3.78-16.46)**	**4.97(2.48-9.95)**
Chemotherapy(Yes vs. No)	1.14(0.70-1.88)	0.595	1.07(0.54-2.13)	1.58(0.71-3.54)
Histological type	1.12(0.80-1.56)	0.511	1.14(0.65-2.01)	1.11(0.71-1.73)

a. HR: Hazards Ratio.

b. CI: Confidence Interval.
